# Sex-Specific Responses of Life Span and Fitness to Variation in Developmental Versus Adult Diets in *Drosophila melanogaster*

**DOI:** 10.1093/gerona/glz175

**Published:** 2019-07-31

**Authors:** Elizabeth M L Duxbury, Tracey Chapman

**Affiliations:** School of Biological Sciences, University of East Anglia, Norwich Research Park, UK

**Keywords:** Longevity, Fecundity, Fertility, Thrifty phenotype, Phenotypic plasticity

## Abstract

Nutritional variation across the lifetime can have significant and sex-specific impacts on fitness. Using *Drosophila melanogaster*, we measured these impacts by testing the effects on life span and reproductive success of high or low yeast content in developmental versus adult diets, separately for each sex. We tested two hypotheses: that dietary mismatches between development and adulthood are costly and that any such costs are sex-specific. Overall, the results revealed the rich and complex responses of each sex to dietary variation across the lifetime. Contrary to the first hypothesis, dietary mismatches between developmental and adult life stages were not universally costly. Where costs of nutritional variation across the life course did occur, they were sex-, context-, and trait-specific, consistent with hypothesis 2. We found effects of mismatches between developmental and adult diets on reproductive success in females but not males. Adult diet was the main determinant of survival, and life span was significantly longer on high yeast adult food, in comparison to low, in both sexes. Developing on a high yeast diet also benefited adult female life span and reproductive success, regardless of adult diet. In contrast, a high yeast developmental diet was only beneficial for male life span when it was followed by low yeast adult food. Adult diet affected mating frequency in opposing directions, with males having higher mating frequency on high and females on low, with no interaction with developmental diet for either sex. The results emphasize the importance of sex differences and of the directionality of dietary mismatches in the responses to nutritional variation.

Dietary quality and quantity are of central importance to fitness and have significant effects on key life-history traits, such as development, life span, and reproductive success (1–8). Hence, responses of individuals to variation in diet across the lifetime are of crucial importance, particularly if there are periods during which each sex exhibits differential nutritional sensitivities. There are two important, and potentially overlapping, facets of nutritional responses: (i) the effects of mismatched diets per se and (ii) the directionality or type of nutritional mismatch. For example, mismatches in the quality or quantity of diets between developmental and adult stages could be crucial determinants of fitness, if the anticipated nutritional environment set during development is not subsequently encountered (9–11). This is the basis of the thrifty phenotype (TP) hypothesis discussed later (9) and, in principle, applies to any type of dietary mismatch encountered across the lifetime. In addition to any dietary mismatching, it is predicted the responses of individuals of each sex will depend on whether a poor versus good diet is initially encountered during development, and how these interact with whatever dietary environment experienced during adulthood. Such effects will depend on the variation in diet quality, the nutritional sensitivity of each sex and the duration of any nutritional stress or surfeit in each life stage (12,13), reviewed by Flatt and Schmidt (14).

A leading idea to explain the effects of dietary mismatching is the TP hypothesis. This proposes that mismatches between developmental and adult environments can influence disease susceptibility and lead to later-life pathologies and reduced fitness (9). Originally derived in the context of human health, its key premise is that beneficial phenotypes expressed in response to developmental conditions (eg, body size and insulin sensitivity) become “fixed” in anticipation of a matching adult environment. If the adult environment is altered (mismatched) from developmental conditions, phenotypes become maladaptive, resulting in life-history costs (9,13,15–17). Despite the prominence of the TP hypothesis, it has hardly been studied in an experimental context.

Though not often explicitly stated, under the TP theory, any kind of dietary mismatching across the life course is predicted to be costly, regardless of its direction and would be evident as a reduction in the life span or reproductive performance of mismatched diet individuals in comparison to those held under more consistent nutritional regimes. However, potential fitness costs of specific types of dietary mismatches can be minimized, which strongly suggests that the directionality or type of dietary mismatch is important, as well as the presence of any mismatch per se. For example, costs of dietary mismatching can be minimized by: (i) a switch from a poor developmental to good adult nutrition leading to compensatory feeding and catch-up growth after the dietary switch, reducing the costs from the “poor start” (2,4,18); (ii) carry-through (silver spoon) benefits (eg, fat reserves) accrued from a nutritionally rich developmental diet to ameliorate a poor adult diet (13); (iii) a harsh developmental environment acting as a filter for developmental viability, selecting only the most resilient individuals, with higher average fitness (reviewed by May and colleagues (5)).

However, an important omission from the theory on the effects of dietary variation across the life course, which we address here, is the existence of significant sex-specific responses to diets (6,19,20). For example, diet has a fundamental (21) and sex-specific (22–26) influence on life span and in general, female life span is more sensitive to diet than is true for males (19). Consistent with this, manipulation of nutrient-sensing genes appears to produce greater extension in female than in male life span (27,28), which predicts that the magnitude of sex differences in life span should vary significantly with diet due to greater life-span plasticity in females. Data from *Drosophila melanogaster* fruit flies support this idea (19), and the resulting corollary that female life span should be more strongly affected by manipulations of nutrient-sensing pathways (29,30). These findings prompt the second key hypothesis we test, that costs and benefits of nutritional mismatches across the life course will be sex-specific. 

Costs and benefits of nutritional mismatching will be shaped by the expression of sex-specific costs and plasticity (5,6,13,14,18,26,31). We can use this to develop a framework to predict how each sex should respond to different directionalities of dietary variation across the lifetime. Males are typically subject to strong selection for reproductive competition and risky reproductive strategies, which promotes investment in secondary sexual traits over the soma (32–34). The fitness benefits for males of reaching a sufficiently competitive body size are potentially large (35) and body size itself exhibits sex-specific optima (36). Large body size is primarily determined by developmental diet in holometabolous insects. Hence, male fitness is expected to be negatively affected by a “poor start” during development (37) because such males will be smaller as adults. Male life span is also relatively insensitive to variation in the adult diet (19), which also suggests that males may not be able to compensate for a poor start even if better diets are encountered during adulthood. However, set against these life-span effects is the strong response of male reproductive success to adult nutrition (38–41), for example, the increased preference for, and reproductive success exposed to, diets high in sugar (42–45). Hence, the ultimate outcomes of nutritional variation on a male’s overall fitness are not yet known. Selection on females, on the other hand, is focused on investment into the adult soma, to build a body that can maximize fecundity during a peak reproductive period (46). Female body size (determined as in males by the developmental diet) shows a strong relationship with fecundity. However, the maintenance of high fecundity depends on a protein-rich adult diet, and both female life span and reproductive success are reported to be highly sensitive to adult diet quality (23,43,44) and much more so than is true for males (19). Female fecundity can “switch” according to the protein in the adult diet within a few hours (47,48). Hence, female reproductive success is expected to be sensitive to developmental diet but have the capacity, unlike males, to recoup additional fitness by rapidly increasing fecundity on a good quality adult diet.

Implicit in the predicted responses of each sex to dietary variation outlined earlier is that life span and reproductive success of males and females should be differentially sensitive. However, such age-specific effects have also not previously been measured (5). With the exception of a study on once-mated females (5) and another on once-mated males and females (6), there has been little work to directly manipulate developmental and adult diets simultaneously and measure the fitness consequences for both sexes. Hence, no study has yet tested, in a fully reproductive, and arguably most realistic, context, for sex-specific fitness effects of dietary mismatching.

Here, we addressed these omissions and tested two specific hypotheses, that (i) dietary mismatches between development and adulthood are costly (9) and (ii) such costs are sex-specific. We experimentally varied the yeast content of developmental (larval) and adult diets in *Drosophila melanogaster*, within a single generation, to create high (H) and low (L) level yeast diets. We created a fully factorial set of combinations across larval and adult stages (HH, HL, LH, LL) and tested the effect on developmental parameters, adult survival, reproductive output, and mating frequency for fully reproductive individuals of both sexes.

## Methods

### Diet Selection

Larval and adult nutritional environments were varied throughout by manipulating only the yeast content of the diet. Low (L; 20 g/L SYA) and high (H; 120 g/L SYA) yeast diets contained either 20% or 120% of the amount of yeast in the standard Sugar Yeast Agar (SYA) diet, but all other dietary components were unchanged (20 g or 120 g Brewer’s yeast, for L and H, respectively, 50 g sucrose, 15 g agar, 30 mL Nipagin, 3 mL Propionic acid per liter). Brewer’s yeast (40.44% protein content) was the primary source of dietary protein and carbohydrate was acquired from both Brewer’s yeast (41.22% carbohydrate content) and from sucrose (sugar, 100% carbohydrate content). The calorie contents of Brewer’s yeast and sucrose are estimated as 3.25 kcal/g and 4 kcal/g, respectively. Calorie and nutrient content calculations were based on published sources (31,49,50). The L diet was therefore 2:5 yeast:sugar; 8.1:58.2 protein:carbohydrate and 265 kcal/L. The H diet was 12:5 yeast to sugar, 48.5:99.5 protein:carbohydrate and 590 kcal/L. Diets were selected based on life span and fecundity curves from (19) that used identical yeast contents and dietary compositions. The L diet was chosen as a stressful, but above starvation, diet. The H diet was selected on the basis that it provided greater nutrition (20% more yeast) than the standard 100 g/L diet, but not to a level that represented “overfeeding.” Four fully factorial diet treatments were set up: LL, LH, HL, and HH (L or H yeast larval then adult diets, respectively). 

### Responses of Survival and Reproductive Success to L and H Diets

The wild-type Dahomey population was used throughout and maintained on standard SYA medium under large population sizes in overlapping generations. Experimental individuals were generated from eggs collected on yeasted red grape agar oviposition plates. A narrow 4 hours egg collection period was used to maximize the resolution of developmental timings. First instar larvae were transferred at a density of 100 larvae per vial to either L or H yeast diets, 26 hours after egg laying. Using developmental timings determined in a pilot study (data not shown), the L diet larvae were set-up 193 hours (8 days and 1 hour) before the H diet larvae, to allow all adults to emerge simultaneously. A separate cohort of control wild-type larvae was reared on standard food at a density of 100 larvae/vial, to generate standard males and females for mating with the focal H and L reared individuals. Analyses of the development time and larval to adult viability data showed the expected differences in development time, with L larvae taking significantly longer to develop and having significantly lower survival to adulthood (Supplementary Figures 1 and 2).

Focal females were mated on their adult treatment diets at 2 days post-eclosion by placing them together with 3- to 4-day-old standard wild-type males for 24 hours. A 60:40 male to focal female ratio was used to introduce a moderate level of male–male competition. Focal males were individually mated with individual, 3- to 4-day-old, standard wild-type virgin females, at a 1:1 ratio, on the focal male adult diet, also at 2 days post-eclosion. Following mating, focal females and males were transferred to individual vials (1 per vial), on the allocated adult diet. Sample sizes were 45 per treatment (LL, LH, HL, HH) for each sex. 

Each individual was exposed for 3 hours every week to a standard, 3- to 4-day-old wild-type mate in a 1:1 ratio. Fresh wild-type flies were generated from cultures every 7 days for these matings. Initial matings occurred at 2 days post-eclosion. All other matings were conducted on the diet of the focal adult. The mating period was limited to 3 hours to minimize responses of the test wild-type females to the different H and L diets on which the males were held. Mating frequency was observed and recorded every 20 minutes during each weekly 3 hours mating test. From this, the weekly proportion of each sex and diet treatment that mated across the lifetime was calculated.

Numbers of focal female and focal male mortalities were recorded daily for each treatment population and each sex. Focal flies were transferred onto fresh food, without CO_2_, every 2–3 days. Weekly 24 hours egg counts were taken from each focal female. Focal males were retained in their mating vials and the wild-type females with which they had mated were transferred into individual vials of standard SYA media to record fecundity over the next 24 hours. Every week focal males were mated for 24 hours with new standard wild-type females, to give, via the fecundity of these females, an estimate of male reproductive output that could be compared with the fecundity of the focal females. Fertility was determined by counts of the first generation of offspring eclosing from the saved egg count vials. Focal female and focal male mortality was recorded daily. 

### Statistical Analyses

All statistical analyses were performed in R, version 3.2.1 (51) and final full models are summarized in Supplementary Table 1.

#### Adult survival

Survival analyses were performed using Cox Proportional Hazards regression analysis, on age-specific mortality data, separately for focal females and focal males. All age-specific mortality data satisfied the proportional hazards assumption of Cox analysis, using both graphical and analytical tests. A Cox model was fitted using the “coxph” function from the “survival” package. Individuals that were lost or died during experimental manipulation were treated as censors in the Cox model. The four diet treatment populations (LL, LH, HL, HH) were partitioned into binary larval and adult diet categorical factors (0 = *low*, 1 = *high protein*) for the analysis. Model simplification was conducted via factor-level reduction from a maximal model including both main effects (larval diet and adult diet) and their interaction to a minimal model containing only significant terms. To test directly for mismatch effects, combined models were specified to test for the effects of the three fixed explanatory factors of interest, namely sex, larval diet, adult diet, and their interaction, using age-specific mortality data from both sexes.

#### Age-specific reproduction

Age-specific egg and offspring counts were analyzed using generalized linear mixed-effects models (“glmer” function from the “lme4” package) to account for the temporal pseudoreplication arising from taking repeated counts from the same individuals over time. The sexes were first analyzed separately and then together using a combined model to test directly for mismatch effects. Poisson error structure was used for count data. Egg count or offspring count was the integer response variable. Larval diet and adult diet and their interaction (larval:adult) were fitted as categorical fixed effects. The number of days post-eclosion on which each count was taken, and a unique identifier assigned to each individual, were both fitted as categorical random factors. Models with the number of days post-eclosion as a fixed effect instead of a categorical random effect gave qualitatively the same results as fitting days post-eclosion as a categorical random factor, for all age-specific reproduction data, but model fit was better with days post-eclosion as a categorical random effect in all cases. The data were overdispersed in all cases. To account for this, an observation level random effect was added to each “glmer” model (the log-normal Poisson distribution) (52,53). Maximum likelihood model comparison showed that this provided best model fit and accounted for zero-inflation in the data set. Egg to adult viability was calculated as the proportion of eggs laid that hatched as viable offspring, at each time point. Proportion data were arcsine transformed and then analyzed with a glmer, with Gaussian errors from the “lme4” package (same output as lmer). Combined models were specified to test for the effects of the three fixed explanatory factors of interest, namely sex, larval diet, adult diet, and their interaction, using age-specific reproduction data from both sexes. Egg count, offspring count, and egg to adult viability data were analyzed separately in the combined models. The differences in age-specific offspring production between mismatched and constant diets, were analyzed with a linear mixed-effects model (lmer) from the “lme4” package. Days post-eclosion and dietary comparison (HL–LL or LH–HH) and their interaction were fitted as fixed effects and the unique identifier for each pair of individuals compared was fitted as a random effect.

#### Lifetime reproductive success

Indices of total lifetime egg production and total lifetime offspring production were calculated separately for each sex and each treatment population by summing weekly 24 hours egg or offspring counts, respectively, across the lifetime, for each individual. Lifetime egg and offspring production data violated the normality and homogeneity of variances assumptions. The Mann–Whitney *U*-test was used to determine the possible significance of pairwise comparisons of treatment levels. Lifetime offspring production was used as a measure of individual-level fitness. A combined model, testing for mismatching, was fitted to test for the effect of sex, diet treatment, and their interaction, on lifetime egg and offspring production, respectively, using a generalized linear model (GLM) with quasipoisson errors.

#### Mating frequency

The proportion of individuals that mated, from each diet treatment population, for each sex and each weekly mating was calculated. An index of mean lifetime proportion mated was calculated from the total number of matings divided by the sum of total number of pairs surviving at each weekly mating over lifetime; for each sex and each treatment population. Mating proportion data were analyzed separately for each sex using a generalized linear model with binomial errors. Overdispersion was accounted for by using quasi-binomial errors. A combined maximal GLM model including larval diet, adult diet, sex, and their interaction was then fitted and stepwise model reduction conducted to determine the minimal adequate model. 

## Results

### Responses of Survival to High and Low Yeast Developmental and Adult Diets

**Figure 1.**
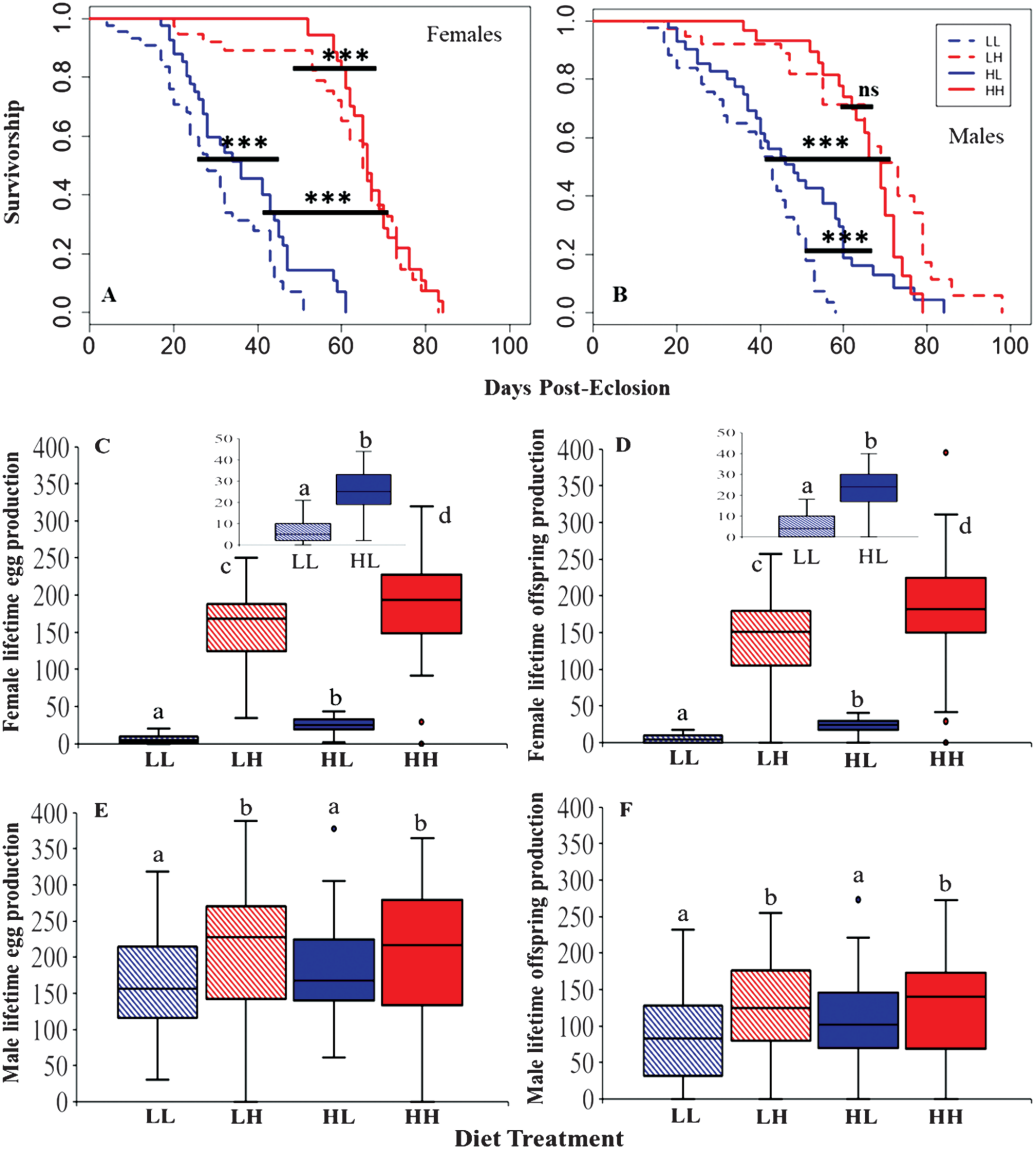
Survivorship and lifetime reproductive success for females and males subjected to LL, LH, HL, and HH diet treatments. LL = constant low yeast (20% SYA); HH = constant high yeast (120% SYA); LH = low yeast larval and high yeast adult diet; HL = high yeast larval and low yeast adult diet. Low or high yeast larval diet (dashed or solid line, respectively) and low or high protein adult diet (blue or red, respectively). Female (**A**) and male (**B**) survival, against time in days since eclosion (*n* = 45 per treatment: median survival of females in days (interquartile range) LL = 27 (18) ,HH = 66 (9), LH = 65 (17), HL = 32 (20); median survival of males in days (interquartile range) LL = 41 (22), HH = 66 (12), LH = 66 (30), HL = 46 (24)). Stars indicate significant differences (*** *p* < .001). Lifetime reproductive success for females (**C** = eggs; **D** = offspring) and males (**E** = eggs; **F** = offspring). Lifetime reproductive success (LRS) for females was calculated from the sum of weekly 24 hours counts of eggs or offspring, for each of the 45 females for each diet treatment. LRS for males was calculated from the sum of weekly 24 hours counts of eggs or offspring produced by standard wild-type (WT) females mated to the focal males, for each diet treatment group. The insets show the LRS for the two low yeast adult diet treatments (LL and HL) in each case. Letters indicate significant differences.

In both sexes, survival was much greater on H than on L adult food and female survival responded more markedly to variation in adult food, as expected, (38 and 34 days difference in median survival for LH–LL and HH–HL females, 25 and 20 days for the corresponding males, respectively). Female survival was determined by main effects of larval and adult diets (coxph: larval diet H > L, *z* = 2.382, *p* < .001; adult diet H > L, *z* = 9.468, *p* = .0172; Figure 1A) and there was no significant interaction. Females raised on H larval food lived significantly longer on both L and H adult diets (ie, HL > LL and HH > LH). In contrast, male survival was determined by an interaction (coxph: *z* = 3.317, *p* < .001), with a significant difference in male survival on the L (HL > LL) but not H (LH = HH) adult diets (Figure 1B); main effects of adult and larval diets were again observed (adult diet H > L, *z* = 6.894, *p* < .001; larval diet H > L, *z* = 3.796, *p* < .001). The patterns of male survival were also replicated in a separate experiment (Supplementary Figure 3A). A combined model analyzing the survival of both sexes simultaneously confirmed a significant sex × larval diet × adult diet interaction effect (coxph regression: *z* = 2.143, *p* = .0321). The results showed a positive carry-over effect of H larval diet onto both adult foods in females, but in males, this occurred for the L adult food only. This revealed that there were no straightforward mismatch costs, but instead sex-specific responses in the extent and pattern of life span to variation in developmental versus adult diets.

### Responses of Reproductive Success to High and Low Yeast Developmental and Adult Diets

#### Age-specific fecundity and fertility

We then conducted analyses of the individual patterns of age-specific fecundity and fertility. This showed that in females, reproductive output was determined by a significant adult × larval diet interaction (glmer egg production: *z* = 4.290, *p* < .001; glmer offspring production: *z* = 3.600, *p* < .001; Supplementary Figure 4A and B) with main effects of both adult and larval diets (glmer egg production: adult H > L, *z* = 23.973, *p* < .001; larval H > L, *z* = 2.424, *p* = .0153; glmer offspring production: adult H > L, *z* = 19.492, *p* < .001; larval H > L, *z* = 2.135, *p* = .0328; respectively). Female reproductive output was always significantly higher on the H adult diet, but the results revealed an additional beneficial carry-over effect of the H larval diet onto both adult diets, with the significant interaction showing that this effect was greater on the H than L adult diet. There was no significant effect of any diet treatment on egg to adult viability (Supplementary Figure 4C). In contrast, in males, adult diet was the sole predictor of reproductive output (egg glmer: *z* = 3.267, *p* = .00109; Supplementary Figure 5A; offspring glmer: *z* = 4.162, *p* < .001; Supplementary Figure 5B), with H adult diet males (HH = LH) having higher egg and offspring production overall than those on the L (LL = HL). Both adult (H > L) and larval (H > L) diets had significant main effects on egg to adult viability (glmer: *t* = 2.987, *df* =1, *p* = .00154; *t* = 1.847, *df* = 1, *p* = .0311; Supplementary Figure 5C). These patterns were again replicated in the additional male experiment (Supplementary Figure 6A–C). A combined model of age-specific reproductive success across both sexes revealed a significant sex × larval diet × adult diet interaction effect on reproductive output (egg glmer: *z* = 2.909, *p* = .00362; offspring glmer: *z* = 2.429, *p* = .0151), but not on egg to adult viability (glmer: *t* = 1.374, *df* = 1, *p* = .167). This showed no main mismatch effects but that there were sex-specific responses in reproductive output to variation in larval versus adult diets. Hence the pattern of sex specificity contrasted with that observed for survival.

#### Lifetime reproductive success

Combining reproductive success across time for individuals allowed us to analyze lifetime reproductive success (LRS; Mann–Whitney U output; Supplementary Tables 2 and 3). Lifetime egg and offspring production were both, as expected, significantly greater for H versus L adult diet females (Mann–Whitney U-test: “eggs” and “offspring” *p* < .001; Figure 1C and D). As for the age-specific reproductive success data, a beneficial carry-over effect of the H larval diet was again apparent with LRS being significantly higher for HL than LL females (Mann–Whitney U-test: “eggs” and “offspring,” *p* < .001) and higher in HH than LH females (Mann–Whitney U-test: “eggs,” *p* = .011; “offspring,” *p* = .007). As in females, males on the H adult diet had significantly higher LRS than those on L (Mann–Whitney U-test: “eggs,” *p* = .004; “offspring,” *p* = .010), but there were no significant differences within those, with HL = LL (Mann–Whitney U-test: “eggs,” *p* = .265; “offspring,” *p* = .0651) and LH = HH (“eggs,” *p* = .753; “offspring,” *p* = .981; Figure 1E and F). These patterns were also found in the replicated males experiment (Supplementary Figure 3B and C). Hence, the beneficial carry-over effects of the H larval diets observed in females were not observed in males. Reflecting this, a combined model including both sexes revealed a significant interaction between sex and diet treatment (lifetime egg production GLM: *t* = 10.419, *p* < .001; lifetime offspring production GLM: *t* = 8.563, *p* < .001). Overall, the results confirm the existence of sex-specific responses of reproductive output to variation in larval versus adult diets.

#### Effect of mismatched diets on age-specific offspring production

**Figure 2.**
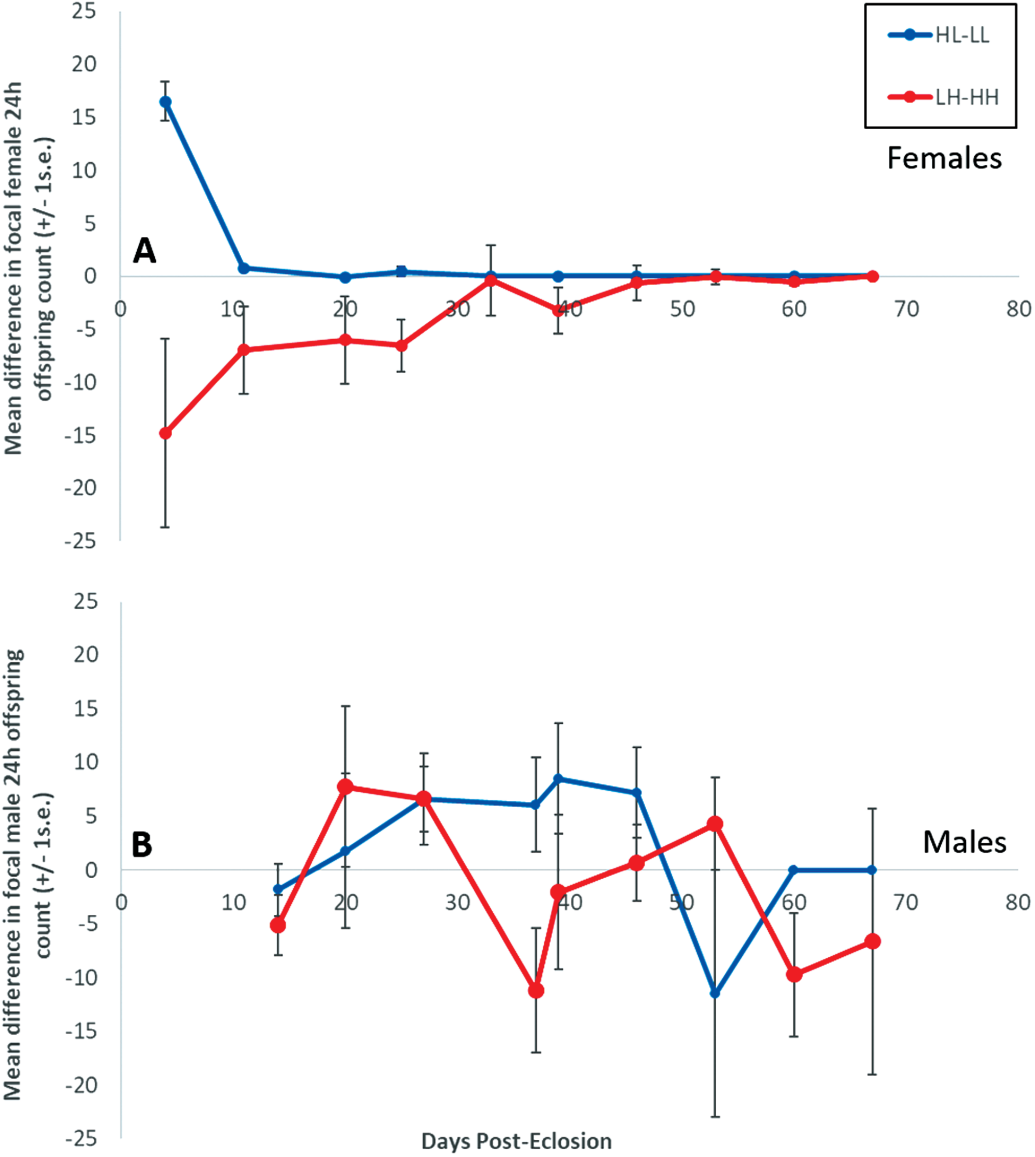
Mean (±*SE*) difference in offspring production per 24 hours between mismatched (HL or LH) and constant (LL or HH) diet treatments, for weekly mated focal females (**A**) and males (**B**), against days post-eclosion. Initial sample size, *n* = 45 for each of the LL, LH, HL, and HH diet treatments. Positive differences indicate higher fecundity on mismatched diet treatments and negative differences lower mismatched diet fecundity when tested on an adult diet of L (blue) or H (red) yeast.

We also tested directly the effects of dietary mismatches by taking the difference in offspring production by males and females kept on constant versus mismatched diets on each adult food type (Figure 2). This highlighted a marked sex difference, with clear effects of mismatches in females but not males. The effect of mismatched diets on females was opposite in direction (significant interaction between adult diet and age; lmer: *t* = 4.764, *p* < .001). HH females had higher reproductive success than LH, suggesting a cost of dietary mismatching, whereas on the L adult diet the pattern was HL > LL, indicating a strong beneficial carry over effect of the mismatched diet. In males, there were no such effects (lmer: “age,” *t* = 0.046, *p* = .934; “adult diet,” *t* = 0.756, *p* = .440; “age” × “diet,” *t* = 1.180, *p* = .233).

### Responses of Mating Frequency to High and Low Yeast Developmental and Adult Diets

Mating frequency exhibited opposing patterns across males and females (Table 1). A combined model of mating frequency across both sexes confirmed a significant interaction between sex and adult diet (GLM: *z* = 3.889, *p* < .001). In males, mating frequency was always higher on the H adult diet and for females, the opposite occurred. The results reveal that the pattern of mating frequency also showed significant sex specificity in response to manipulations of the larval versus adult diet, but that, again, the pattern of responses contrasted with that seen for life span or reproductive success.

**Table 1.** Mean Mating Frequency Index Over The Lifetime, for Weekly Mated Focal Females and Males Across Diet Treatments (LL, LH, HL, HH)

	LL	LH	HL	HH
Females	0.66	0.59	0.64	0.58
Males	0.84	0.91	0.84	0.89

Index of mean mating frequency was calculated as the number of focal individuals for each diet treatment and each sex mating each week, as a proportion of the total number of surviving pairs, summed over the lifetime.

## Discussion

The results showed that, in a biologically relevant, fully reproductive context, that there were no simple costs of mismatched diets. Instead, we observed sex-, trait-, and context-specific responses to dietary variation across the lifetime. These were evident as significant sex differences in how male and female life span, reproductive success and mating frequency responded to variation in developmental versus adult diets. Rather than clear evidence for costs of mismatching, it was the directionality of mismatching, or not, that determined male and female fitness. In general, females showed greater responsiveness than did males to adult diets and there was evidence for positive carry-over effects of a good larval environment into adult female survival and reproductive success. The results highlight the complexity of nutritional responses and show that a consideration of the divergent responses of each sex is essential in order to predict fitness outcomes.

### Costs of Dietary Mismatches Between Development and Adulthood

The TP theory suggests that mismatched diets are expected to be costly because individuals are “set” on a nutritional path during development that then fails to be realized. In this sense, any kind of mismatching between developmental and adult diets would be expected to be costly (9,15). This hypothesis was not borne out here, as mismatched diets were sometimes costly, sometimes not, depending on the direction of the developmental to adult mismatch and the sex in which it occurred. Instead, the strongest determinant of survival, reproduction, and mating frequency was not match or mismatch, but the yeast content of the adult diet. For both sexes, the H adult diet always produced individuals with higher survival, reproductive output, and fitness. This was expected on the basis that we selected the high yeast diet to represent good quality and the low to be stressful (19,21,41,54). Diet treatments that went from high to low actually improved the survival of both sexes, whereas switching from low to high diets did not. As for survival, female reproductive success was also higher in the mismatched treatment when the switch was from high to low but with the opposite pattern occurring in the low to high diet treatment, with no such effects observed in males. This revealed a lack of support for general costs of mismatching but instead, the existence of significant context dependence (benefits and costs depending upon whether switches are from high to low, or low to high protein diets) that was different for each sex, as discussed further later. In general, the results suggested that beneficial carry-over effects from a good quality developmental diet can over-ride costs of developmental to adult dietary mismatches.

### Directionality of Dietary Mismatching (Poor Start and Silver Spoon Effects)

A good start or “silver spoon” dietary environment (H larval diet) was beneficial to female survival and reproductive success regardless of the type of diet encountered during adulthood. In contrast, male survival benefited from a good start only when adult diets were poor, and there was no overall effect of a good start on male reproductive success. This suggests that female survival and reproductive success is more sensitive to silver spoon effects than is true for males. For males, the primary determinant of mating success was the high adult diet, regardless of developmental diet. This suggested significant effects of adult diet quality on male mating success that were separate from effects of diet on survival and LRS. Effects of adult diets on focal male courtship, mating, and the quantity and quality of sperm production are well known (38–41). However, the effect of adult diet on male mating behavior can be variable. For example, Fricke and colleagues (40) showed that males on a low protein adult diet had reduced successful courtship (for re-matings with non-virgins), which is consistent with the reduced mating success observed here. Furthermore, a low protein larval diet is known to reduce the quantity of sperm males transfer to females, which may affect its viability (39). However, Fricke and colleagues (41) found no difference between male courtship on low versus high-protein adult diets. These results show that different traits that contribute to male fitness can show complex and sometimes opposing responses to adult diet quality. These effects require further study.

### Sex-Specific Costs and Benefits of Dietary Mismatching

In terms of survival, males and females that had a good start (H developmental food) and then encountered poor adult (L) food showed increased survival. The survival of females, but not males, was also increased in individuals that consistently encountered high yeast food across their lifetimes. Individuals held on high adult diets generally achieved significantly greater LRS than on low. However, there were again significant sex-specific responses, with females, but not males, benefitting from positive carry-over effects of a high yeast larval environment into a low yeast adult environment. There were no carry-over effects or costs/benefits of dietary mismatching for mating success, but instead a strong and opposing effect of adult diet, with male mating frequency being elevated on a high yeast diet in adulthood, with the opposite effect being found in females.

Overall, our findings do not provide strong or consistent support for the TP theory (9) as they suggest that a high larval diet can ameliorate the costs of a mismatched low adult diet. Though there were benefits of a consistent high-quality diet across the whole lifetime for female survival (which would support the TP hypothesis), such effects were neither seen in males nor evident on the consistently low-quality diet. A poor quality maternal diet during development has been shown to reduce offspring production in other species, despite a good quality maternal diet in adulthood, perhaps suggesting that such mechanisms are conserved (55). Our findings are also in agreement with previous studies that found no adult life-span effects due to restricted larval diets (56,57). The subtle effects we observed were in line with the finding that delayed maturation, and longer development time on low protein diets, is associated with increased longevity (eg, in the house cricket, *Acheta domesticus* (58–60). The existence of male-specific fitness benefits of a mismatched low protein larval diet followed by a good quality adult diet may have arisen as a consequence of the low protein larval diet acting as a stronger developmental viability selection filter on male than female fitness. This possibility should be investigated further.

Our results are consistent with previous reports of sex-specific effects of nutrition on reproduction (5,23). It would also be interesting to explore further the impact of good and poor larval diets in an ecological context. For example, it would be informative to discover the extent to which effects of larval diet quality on development speed and viability affect physiological resilience to disease or stresses experienced during adulthood and to test for nutritionally sensitive periods (61). A good larval diet always increased developmental rate and viability, in line with published data (5,56,57). The puparium to adult stage of development appeared to be most sensitive to the detrimental effects of an L larval diet, as would be expected on the basis of the energetically expensive developmental remodeling that occurs during this life stage (13).

## Conclusions

This study tested the hypotheses that dietary mismatches between development and adulthood are costly and that such costs are sex-specific. The results showed a mixture of support for these ideas. There was no consistent support for costs of dietary mismatches, which were only sometimes observed. However, costs and benefits of dietary mismatches between development and adulthood did show significant sex specificity and contrasting patterns across survival and reproductive success. Female survival and reproductive success did benefit from a good start more so than was true for males. However, male mating success was independent of a good or poor start when encountering a good diet in adulthood. The results supported the prediction that the sexes have specific responses to dietary variation and that costs and benefits varied across the lifetime. Hence, not all phenotypes expressed in response to the developmental environment are “set” and significant life-history plasticity was evident in the responses of individuals to matched or mismatched adult dietary environments. This is consistent with the previously observed rapid plasticity to short-term dietary manipulation within adult life (14,31). Hence, under conditions where mismatches are common (eg, fluctuating environments), there could be selection to counter the potentially deleterious effects of mismatches. These findings add to the body of evidence to show how nutrient regimes affect male and female survival (19,21,62–64). They highlight the importance of measuring multiple life-history traits in both sexes and justify further investigations of the life history consequences of nutritional mismatches and the factors that can ameliorate the costs of mismatches over a broader range of diets (62) or over a cross-generational time-scale.

## Supplementary Material

glz175_suppl_Supplementary_Material
