# Supplementary material for: Sex-Specific Responses of Life Span and Fitness to Variation in Developmental Versus Adult Diets in Drosophila melanogaster
Source: J Gerontol A Biol Sci Med Sci. 2019 Jul 31;75(8):1431–8. doi: 10.1093/gerona/glz175 (PMC7357588; doi:10.1093/gerona/glz175)
Supplement: glz175_suppl_Supplementary_Material [file glz175_suppl_supplementary_material.pdf]

Sex-Specific Responses of Lifespan and Fitness to Variation in Developmental versus Adult Diets in *D. melanogaster*

Elizabeth M.L. Duxbury and Tracey Chapman

School of Biological Sciences, University of East Anglia, Norwich Research Park, NR4 7TJ, UK.

Supplementary Material

(i) Development time and viability of individuals raised on low and high food developmental diets – methods and results. Figure S1, S2.

(ii) Replicated male experiment – methods, results, survival and lifetime reproductive success. Figure S3.

(iii) Age-specific fecundity and fertility for females and males in the main experiment. Figure S4, S5.

(iv) Replicated male experiment – age specific fecundity and fertility. Figure S6.

(v) Full final model codes for statistical analysis of survival, reproduction and mating frequency in the main experiment. Table S1.

(vi) Female lifetime reproductive success – statistical analysis. Table S2.

(vii) Male lifetime reproductive success – statistical analysis. Table S3.

(i) Development time and viability of individuals raised on low and high food developmental diets

Methods: Assays of development time and developmental survival were conducted in a separate experiment, on L or H SYA food medium. Experimental individuals were generated from eggs collected on yeasted red grape agar oviposition plates, from a large population cage of wild type flies. Laboratory-caged flies had been reared on standard (100% SYA) food for multiple overlapping generations, at 25°C, 50% relative humidity and a 12:12h light:dark cycle, since the 1970s. Eggs were collected over a narrow period of 4h, to facilitate precise monitoring of subsequent developmental timings. Larvae were picked from the plates 26h after oviposition and transferred to vials of either L or H protein food at a density of 100 larvae/vial. 10 vials of each food type were set up. Numbers of puparia were recorded twice daily from Day 5 onwards. The number of eclosed adults from the same larval vials were also recorded twice daily, until Day 28. From these measurements we determined egg to pupariation and egg to adult duration times. These data were expressed as proportion data and analysed using a generalised linear model (GLM) with quasi-binomial errors to account for overdispersion. Differences in the number of eclosed adults per sex per replicate vial were analysed using a paired t-test, separately for each diet treatment (normality and equality of variances assumptions were met). Development time data were tested for normality with the Shapiro Wilk test and for equality of variances with the Levene's test. Differences in development time between diet treatments were analysed using the two sample t-test, where the assumptions were met, and the Welch two sample t-test, when variances were unequal.

Results: Developmental viability was significantly higher on the H in comparison to L larval diet, for overall egg to adult viability (GLM: $t = 3.907$, $p = 0.00103$; Figure S1a) and for puparium to adult (GLM: $t = 3.796$, $p = 0.00132$; Figure S1c). There was no significant difference in egg to puparium viability (GLM: $t = 1.378$, $p = 0.185$; Figure S1b). These viability results were independently replicated, for egg to adult (e-a), puparium to adult (p-a) and egg to puparium (e-p) stages (GLM: $t=12.95$, $p<0.001$, for e-a; $t=13.01$, $p<0.001$, for p-a; $t=1.966$, $p=0.0586$, for e-p; data not shown). As expected, development time was significantly longer on the L in comparison to H larval diet, for overall egg to adult development time (Two

Sample t-test: t=30.825, d.f.=9.807, p<0.001; Figure S2a), egg to puparium (t=32.803, d.f.=9.084, p<0.001; Figure S2b) and puparium to adult (t=5.0815, d.f.=11.079, p<0.001; Figure S2c) stages.

Figure S1

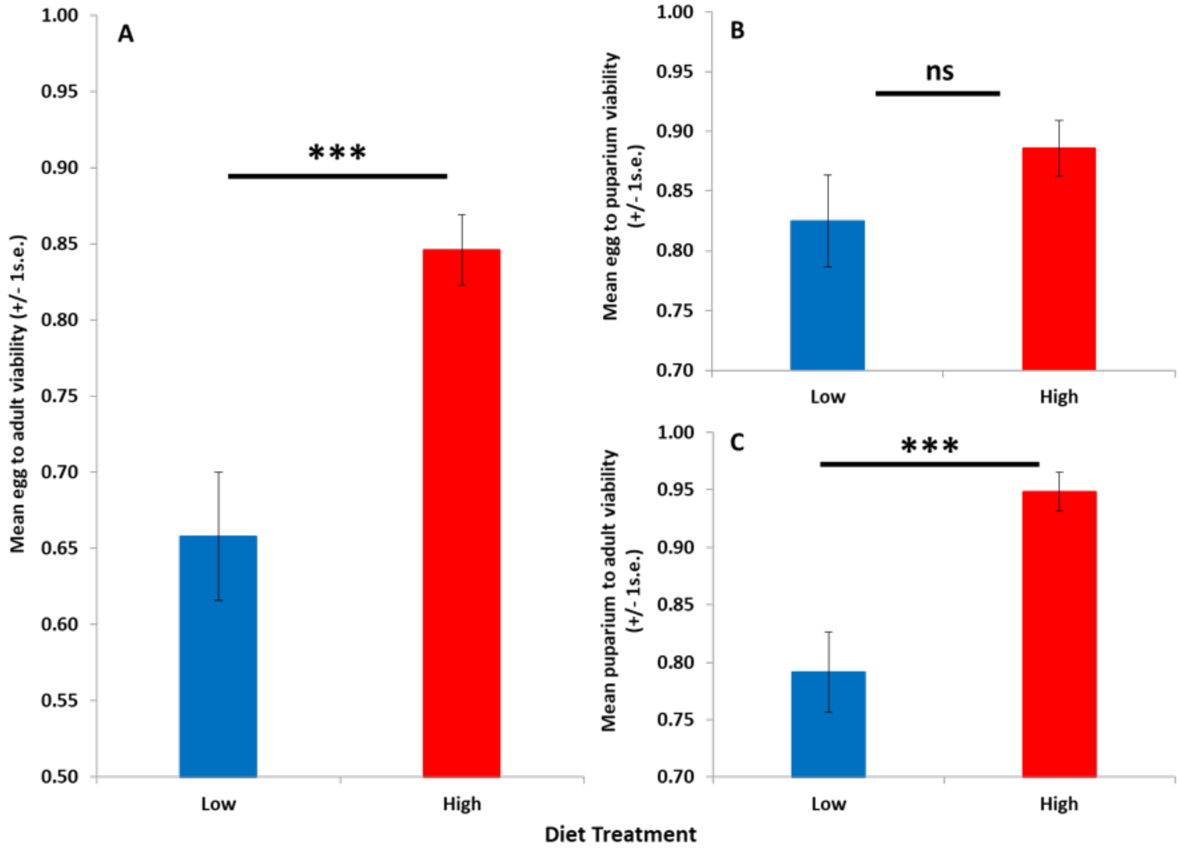

Figure S1. Mean developmental viability (+/- 1s.e.) on low (20g/L) or high (120g/L) yeast larval diets, at egg to adult (A), egg to puparium (B) and puparium to adult (C) developmental stages. Sample size of 10 vials each containing 100 larvae per diet treatment. Stars indicate significant differences (*** $p < 0.001$).

Figure S2

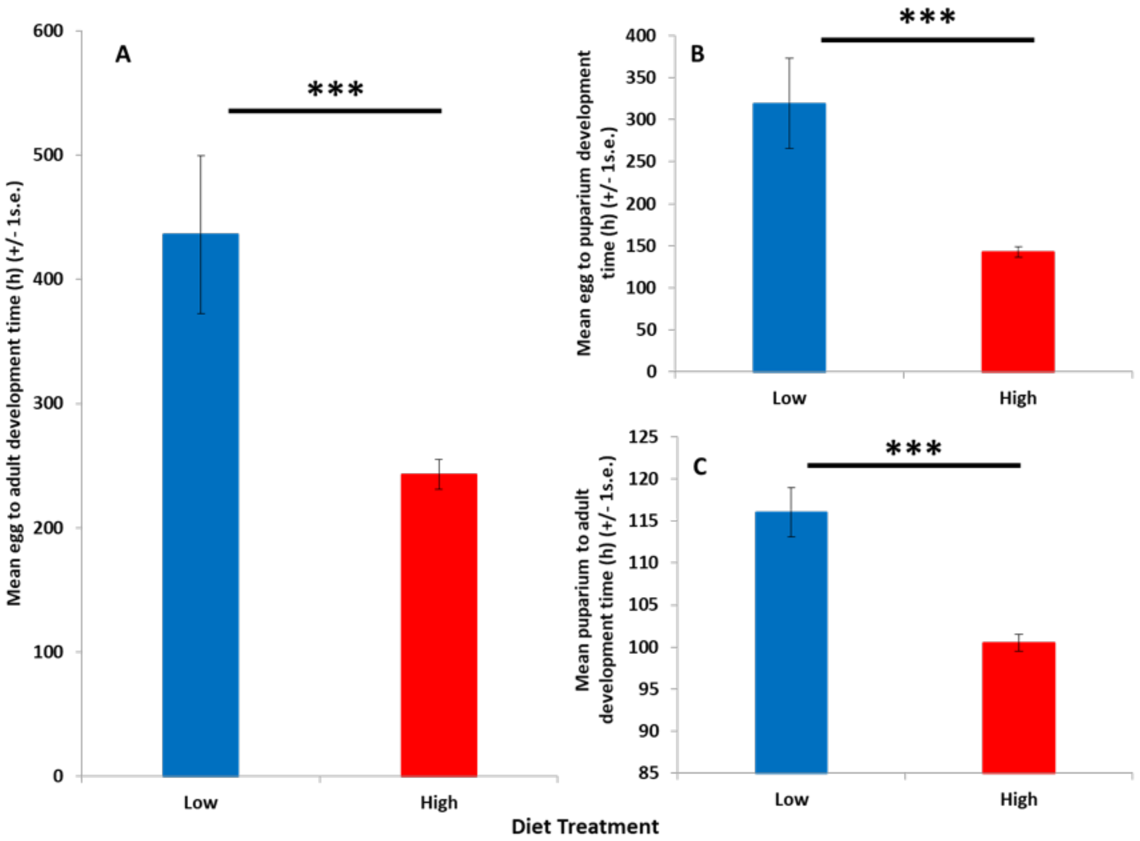

Figure S2. Mean development times (+/- 1s.e.) on low (20g/L) and high yeast (120g/L) larval diets, at egg to adult (A), egg to puparium (B) and puparium to adult (C) developmental stages. N=10 vials of 100 larvae per diet treatment. Stars indicate significant differences (*** $p < 0.001$).

(ii) Replicated male experiment

A replicated experiment on male survival and reproductive success was conducted, to test for replicability of the patterns of survival and reproductive success. The methods were exactly the same as per the main experiment described in the main text, except that focal males were mated with wild type females, once per week, for 24h rather than 3h and a sample size of 35 individually housed males per treatment were assayed.

Results

Survival. Male survival was determined by a significant interaction between adult and larval diet (z=2.81, p=0.005), with a significant difference in survival on the L (HL > LL) but not H (LH = HH) adult diet treatments (Figure S5). This gave evidence for a positive carry-over male survival effect of H larval diet onto L adult food. Main effects of adult (H > L; z=7.67, p<0.001) and larval (overall H > L; z=2.66, p=0.008) diets on male survival were also observed.

Age-specific fecundity and fertility: Fecundity in the mates of fully reproductive males was determined by an interaction between adult and larval diet (glmer:z=1.96, p=0.050), with main effects (H > L) of adult diet on egg (z=14.419, p<0.001; Figure S6A) and progeny production (z=13.663, p<0.001; Figure S6B). HH males had consistently higher fecundity than LH males and fecundity and offspring production was greater on the H than L adult diets. There was no larval x adult diet interaction or main effect of larval diet on offspring production. Only larval diet had a significant effect on egg to adult viability (glmer: t=2.481, d.f.=1, p=0.0146; Figure S6C). Males experiencing a poor start and then a good adult diet had lower reproductive success than did males with a consistently good diet throughout life. There were no carry-over effects of good quality larval nutrition on a poor adult diet. Hence, males were less able to compensate for a poor start, in contrast to once-mated females, in terms of reproductive output.

Lifetime reproductive success (LRS): Weekly-mated males on the H adult diet had significantly greater LRS than those held on the L adult diet (M-W U test: lifetime eggs, W=209, p<0.001; lifetime offspring, W=197, p<0.001; Figure S7 A,B). There was no significant difference in lifetime egg or offspring production for HL and LL males (M-W U test: lifetime eggs, W=760, p=0.208; lifetime offspring, W=699,

p=0.572) or between HH and LH diets (lifetime eggs, W=632, p= 0.861; lifetime offspring, W=560, p =0.324; respectively).

Figure S3.

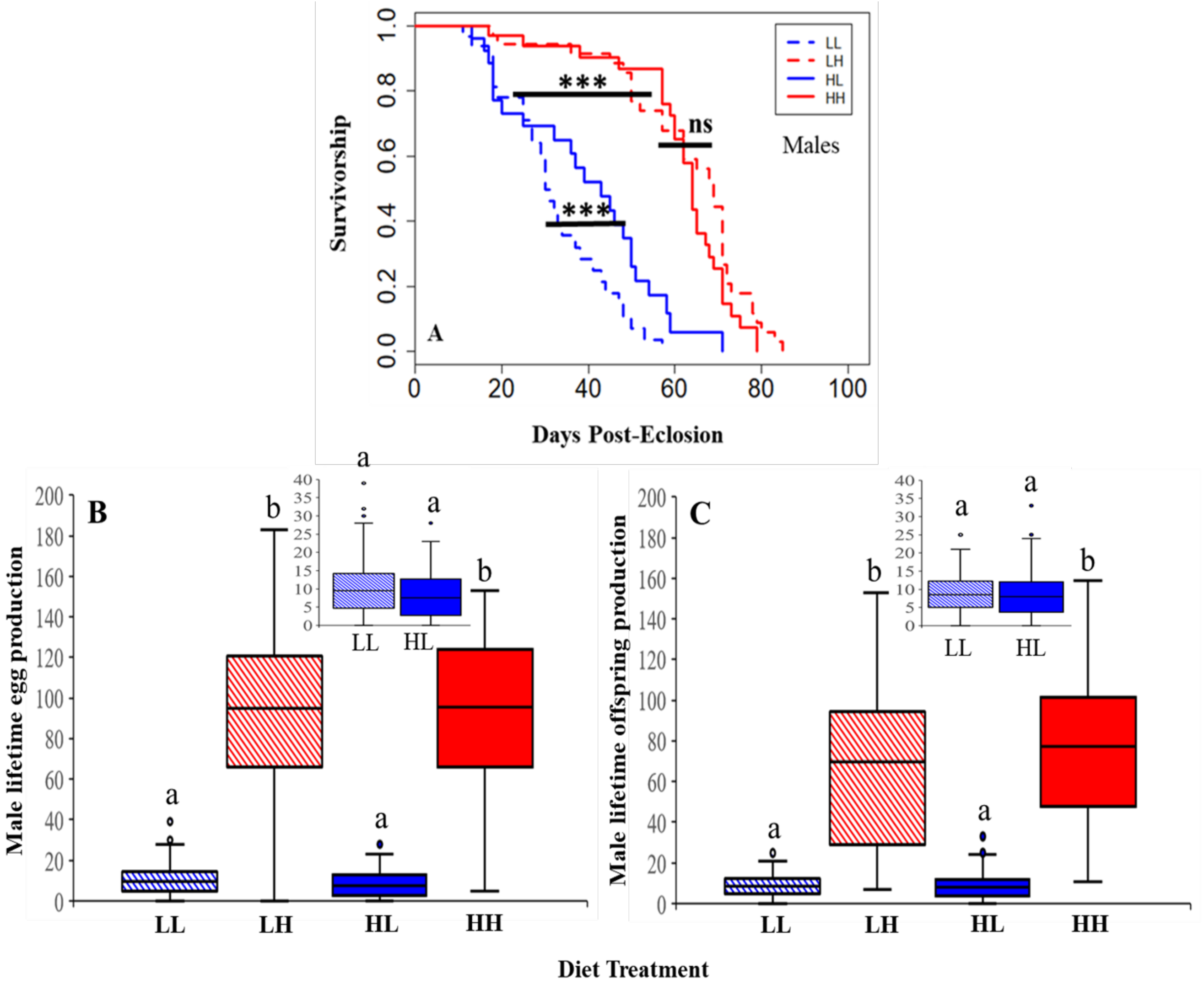

Figure S3. Survivorship and lifetime reproductive success for males subjected to LL, LH, HL and HH diet treatments – replicated male experiment. LL = constant low yeast (20g/L SYA); HH = constant high yeast (120g/L SYA); LH = low yeast larval and high yeast adult diet; HL = high yeast larval and low yeast adult diet. (A) Male survival, against time in days since eclosion (n=35 per treatment: median survival of males in days (interquartile range) LL = 30 (16) ,HH = 64 (11), LH = 69 (19), HL = 39 (31). (B, C) Lifetime reproductive success for males calculated from the sum of weekly 24h counts of eggs (panel B) or offspring (panel C) produced by standard WT females mated to the focal males, for each diet treatment group. The insets show the LRS for the two low yeast adult diet treatments (LL and HL) in each case. Letters indicate significant differences.

(iii) Age-specific fecundity and fertility for females and males - main experiment

Figure S4

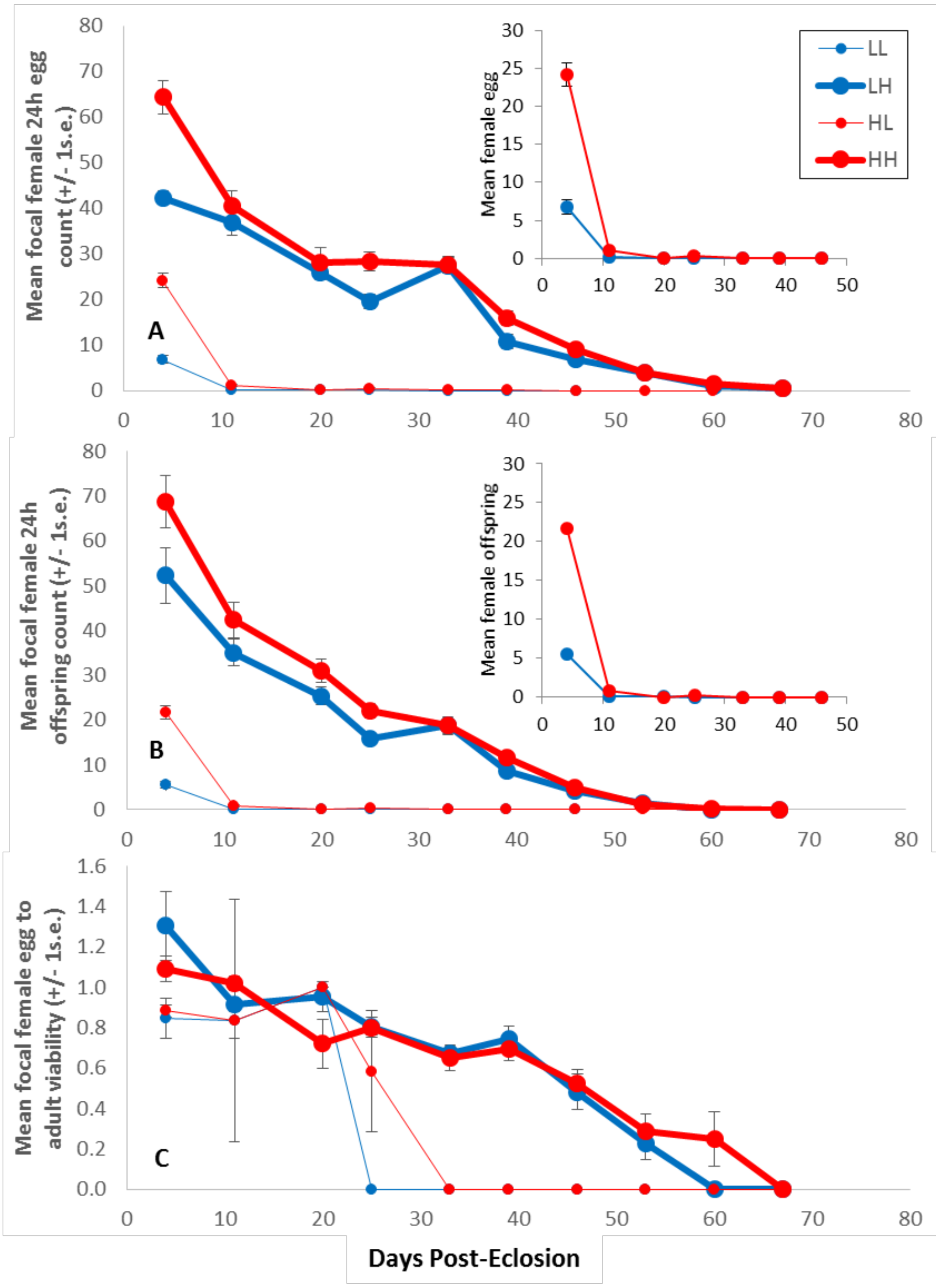

Figure S4. Age-specific egg (A) and offspring production (B) and egg to adult viability (C), (mean ±1se) per female, per 24h, against days post-eclosion, for focal females (mated each week with standard wild type males) in the main experiment. Initial sample size, n=45 females for each of the LL, LH, HL and HH diet treatments (L = low (20g/L) yeast; H = high 120g/L% yeast). Egg to adult viability is the proportion of eggs which eclosed as adults. Insets on panels (A) and (B) show egg and offspring counts for the two L yeast adult diet treatments: HL (red) and LL (blue).

Figure S5

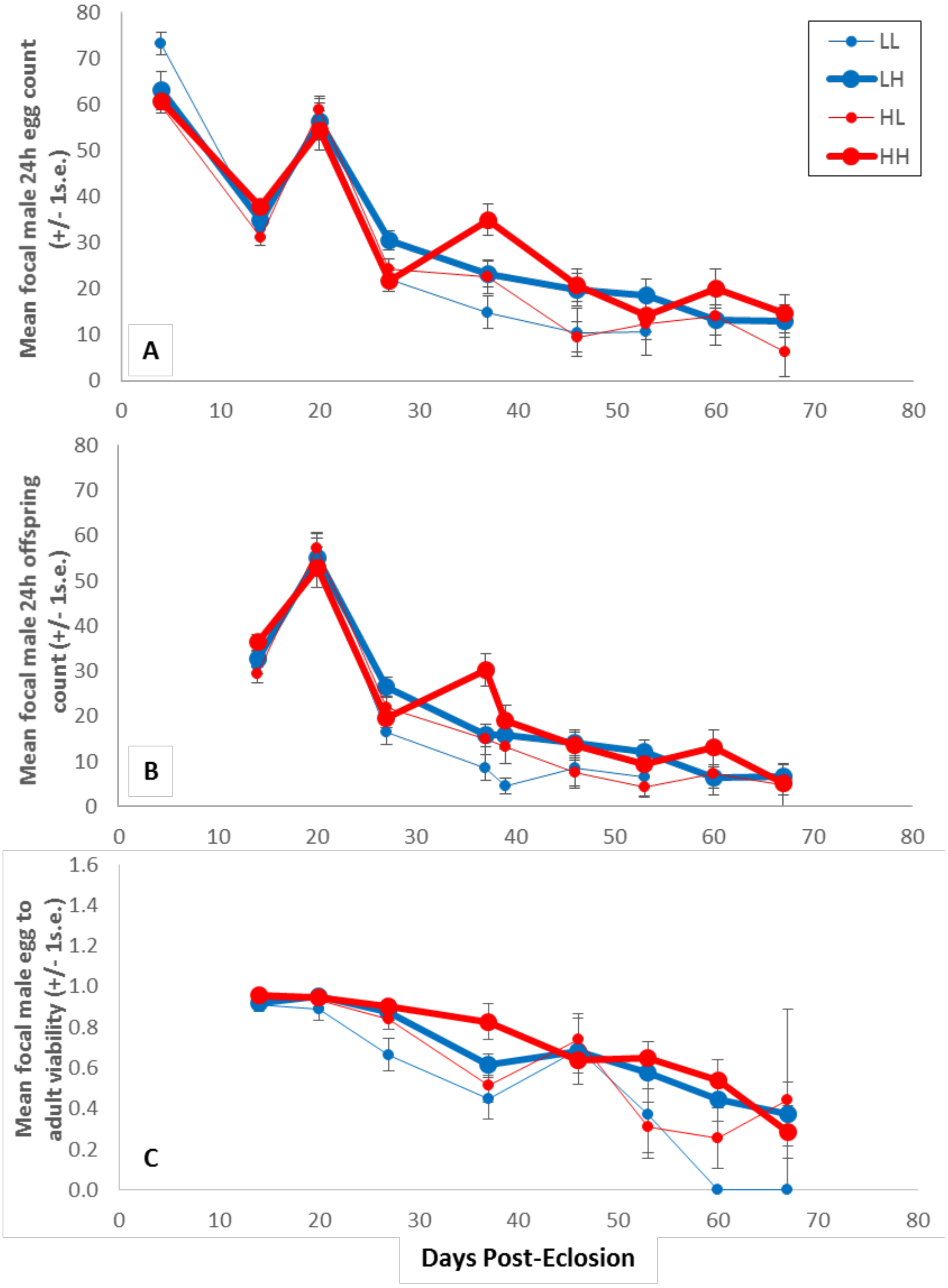

Figure S5. Age-specific egg production (A), offspring production (B) and egg to adult viability (C) (mean ±1se) per 24h against days post-eclosion for focal males (mated each week with standard wild type females) in the main experiment. Initial sample size, n=45 males for each of the LL, LH, HL and HH diet treatments (L = low (20g/L) yeast; H = high 120g/L yeast). Egg to adult viability is the proportion of eggs laid by the wild type females that eclosed as adults (C).

Figure S6

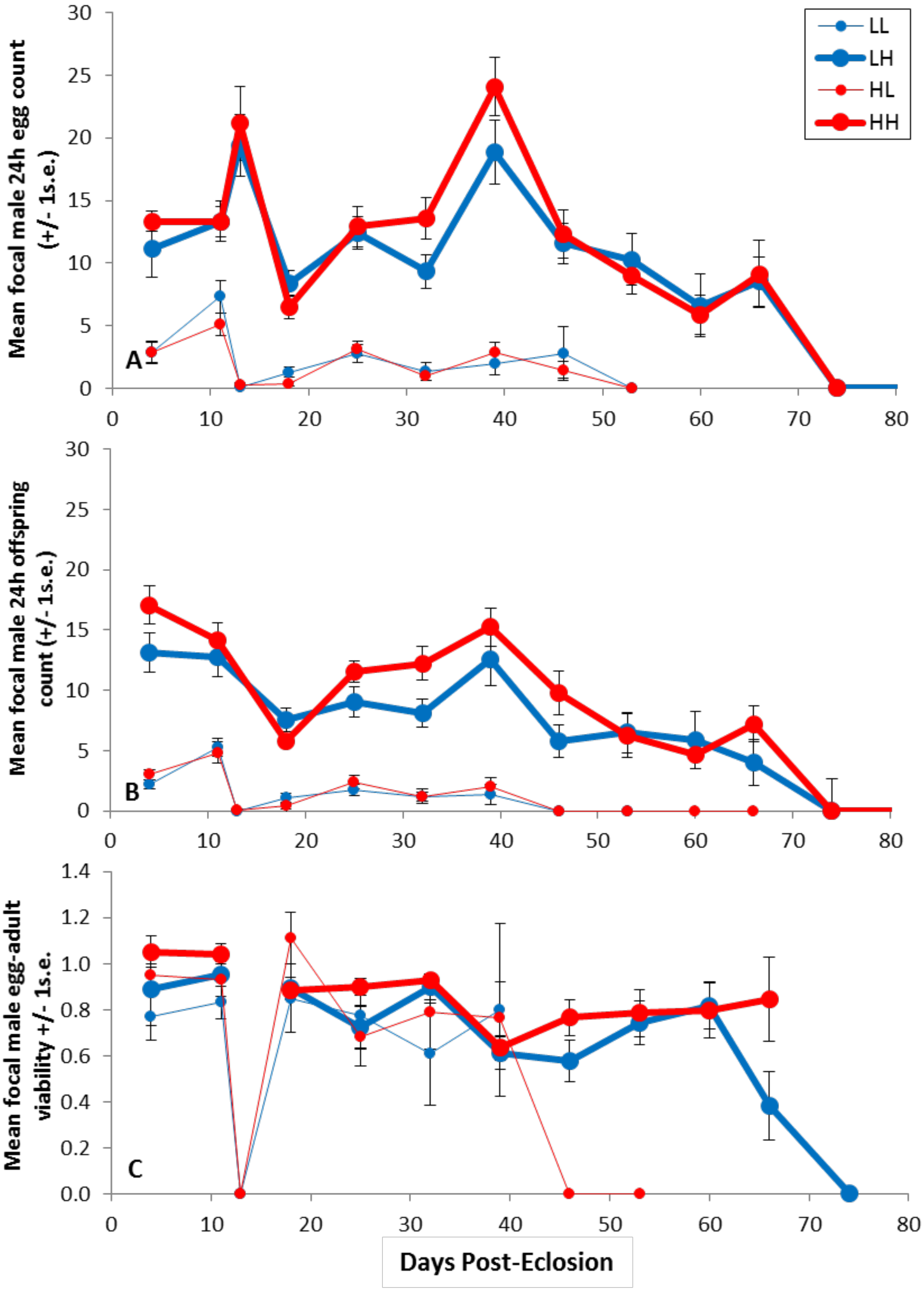

Figure S6. Age-specific egg production (A), offspring production (B) and egg to adult viability (C) (mean ±1se) per 24h against days post-eclosion, for focal males (mated each week to standard wild type females) in the replicated male experiment. Initial sample size, n=35 males for each of the LL, LH, HL and HH diet treatments (L = low (20g/L) yeast; H = high 120g/L yeast). Egg to adult viability is the proportion of eggs laid by the standardised wild type females that eclosed as adults.

Table S1. Full final model codes for statistical analysis of survival, reproduction and mating frequency. Survival analyses were performed on age-specific mortality data, using Cox Proportional Hazards regression analysis. Status indicated whether individuals died or were censored. Age-specific reproduction and egg to adult viability were analysed using generalised linear mixed effects models and the differences in offspring production between mismatched and constant diets were analysed with linear mixed effects models. Lifetime reproduction data was analysed with a generalised linear model (GLM) with quasipoisson errors. Mating frequency was analysed with a GLM with binomial errors. Combined analysis included data from both sexes. Final full models contain only significant factors, following factor-level reduction from a maximal model containing all main effects and their interaction (indicated by ":").

**Life history trait**	**Final Full Model**
Females	
Survival	(age.at.death,status)~larval+adult
Age-specific reproduction	egg.count~larval+adult+larval:adult+(1|age)+(1|individual)
	offspring.count~larval+adult+larval:adult+(1|age)+(1|individual)
	offspring.count.difference~adult.diet+age+adult.diet:age+(1|pair)
	egg.adult.viability~1+(1|age)+(1|individual)
Males	
Survival	(age.at.death,status)~larval+adult+larval:adult
Age-specific reproduction	egg.count~adult+(1|age)+(1|individual)
	offspring.count~adult+(1|age)+(1|individual)
	offspring.count.difference~age+(1|pair)
	egg.adult.viability~larval+adult+(1|age)+(1|individual)
Combined analysis	
Survival	(age.at.death,status)~larval+adult+sex+larval:adult:sex
Age-specific reproduction	egg.count~larval+adult+larval:adult+sex+(1|age)+(1|individual)

	offspring.count~larval+adult+larval:adult+(1|age)+(1|individual)
Lifetime reproduction	lifetime.egg.count~sex+diet.treatment+sex:diet.treatment
	lifetime.offspring.count~sex+diet.treatment+sex:diet.treatment
Mating frequency	proportion.mating~ adult.diet+sex+adult.diet:sex

Table S2. Pairwise comparisons of female lifetime reproductive success (LRS) for weekly-mated focal females across diet treatments (LL, LH, HL, HH). LRS was calculated as the total egg or offspring production by each focal individual over lifetime. The Mann-Whitney U test was used to determine the possible significance of pairwise comparisons of high (H) or low (L) larval then adult diets.

**Comparison**	**W**	**p**
Female lifetime egg production		
H vs L adult diet	117	<0.001
HL vs LL	135	<0.001
HH vs LH	696	0.011
Female lifetime offspring production		
H vs L adult diet	196	<0.001
HL vs LL	164	<0.001
HH vs LH	680	0.007

Table S3. Pairwise comparisons of male lifetime reproductive success (LRS) for weekly-mated focal males across diet treatments (LL, LH, HL, HH). LRS was calculated as the total egg or offspring production by each focal individual over lifetime. The Mann-Whitney U test was used to determine the possible significance of pairwise comparisons of high (H) or low (L) larval then adult diets.

**Comparison**	**W**	**p**
Male lifetime egg production		
H vs L adult diet	3046	0.004
HL vs LL	874	0.265
HH vs LH	1052	0.753
Male lifetime offspring production		
H vs L adult diet	3150	0.010
HL vs LL	784	0.065
HH vs LH	1016	0.981
